# Processes of change, pros, cons, and self-efficacy as variables associated with stage transitions for effective stress management over a month: a longitudinal study

**DOI:** 10.1186/s40359-022-00822-8

**Published:** 2022-05-12

**Authors:** Ke Deng, Akira Tsuda, Satoshi Horiuchi, Shuntaro Aoki

**Affiliations:** 1 Institute of Comparative Studies of International Cultures and Societies, Kurume University, Fukuoka, Japan; 2 Graduated School of Medical Sciences, Teikyo University of Science, Tokyo, Japan; 3 Department of Social and Clinical Psychology, Hijiyama University, Hiroshima, Japan; 4 Center for Medical Education and Career Development, Fukushima Medical University, Fukushima, Japan; 5 Department of Neuropsychiatry, Fukushima Medical University, Fukushima, Japan

## Abstract

**Background:**

The transtheoretical model of intentional health behavior change categorizes people into experiencing five stages in understanding the process of initiating and maintaining effective stress management (i.e., engagement in any form of healthy activity that is practiced for at least 20 min per day). The first purpose of this study was to observe whether any cases would disclose stage misclassification over one month. The second was to examine whether different model's variables are associated with the stage transitions for effective stress management at different stages.

**Methods:**

Data from 946 Chinese students and workers were subjected to analyses. This study is a part of a larger, longitudinal web-based study in which three surveys were conducted in March, April, and September 2014. This study analyzes the data of demographic variables, perceived stress, stages of change, processes of change, pros, cons, and self-efficacy at the point of the first survey and stages of change at the point of the second survey.

**Results:**

Of 144 participants who progressed from the pre-Action stages to the post-Action stages, 44 then progressed to Maintenance (practicing effective stress management for six months or longer). These patterns could not technically occur, and thus, these participants were excluded from the following analyses. Data from the remaining 902 participants were subject to a series of logistic regression analyses. Generally, the model’s variables failed to predict the stage transitions. Exceptions were found that higher experiential processes (the cognitive activities required to progress through stages) and lower self-efficacy (the confidence that one can engage in effective stress management despite barriers to it) predicted the forward and backward stage transitions from Precontemplation (with no intention to initiate effective stress management in the next six months) and Action/Maintenance (practicing effective stress management).

**Conclusions:**

Evidence of stage misclassification indicated the limitations of the model’s stage classification. Experiential processes and self-efficacy as predictors at different stages were in line with the model’s assumption that different variables are assumed to be predictors of stage transitions at different stages, partially supporting the utility of the stage classification.

## Background

Stress management is an important topic across many countries in the world including China [1, 2]. Stress refers to the relationship between the quality of a potentially stressful situation (stressor) and an individual’s personal ability to cope with the stressor [3]. It can adversely affect health [4] and work productivity [5]. Due to these impacts, stress is markedly prevalent in China, representing one of the nation’s major public health problems [6, 7]. Advancing one’s stress management behavior may serve as one of the first, attractive steps for managing stress. Stress management behaviors are defined as behaviors people often deploy when they face stressors to attempt to manage painful or difficult emotions [8–10]. However, in [11], Deng and Tsuda reported that around half of Chinese students and adults did not deploy such behaviors. The encouragement of an individual’s stress management behavior first requires access to psychological models to understand the process of initiating and maintaining stress management behavior [12]. Such models can be used to develop and evaluate interventions.

To understand the process of stress management behavior change, a multi-theory model [13] and transtheoretical model (TTM) of intentional health behavior change [14] have been deployed. Of these models, the TTM may be the most salient for understanding the process of initiating and maintaining stress management behavior. Based on [11], this study focuses on effective stress management, which is an example of stress management behavior. A working definition of effective stress management is engagement in any form of healthy activity that is practiced for at least 20 min per day. Applied to effective stress management, the TTM describes the process of one’s initiating and maintaining effective stress management as a progressive journey through the following five stages of change [15]: precontemplation (not intending to initiate a program of effective stress management in the next six months); Contemplation (intending to initiate a program of effective stress management in the next six months); Preparation (intending to initiate a program of effective stress management in the next 30 days); Action (practicing a program of effective stress management for less than six months); and Maintenance (practicing a program of effective stress management for six months or longer). The model assumes that stages of change are open to variation to some extent and stable to another extent, but most people remain stuck in earlier stages rather than progressing to later stages without professional interventions [15].

The TTM also encompasses processes of change, pros, cons, and self-efficacy that are systemically related to stages of change [15]. Applied to effective stress management, processes of change refer to covert and overt activities that individuals are encouraged to practice to progress to the next stage and are broadly divided into experiential and behavioral processes. An example of experiential processes is consciousness-raising, which refers to increasing awareness regarding stress management. An example of behavioral processes is stimulus control, which refers to the restructuring one’s environment to facilitate the process of stress management. Pros and cons encapsulate the advantages and disadvantages of effective stress management. Self-efficacy refers to the confidence that the individual can engage in effective stress management processes despite any barriers to that process. Table 1 shows definitions of the model’s constructs. Both pros and self-efficacy are assumed to show patterns of increases while cons show those of decreases with stage advancement. Although more research is needed, generally, experiential processes of change are more important in the Precontemplation and Contemplation stages and behavioral processes are important in the Preparation and Action stages [15]. The model assumes that different processes of change, pros, cons, and self-efficacy are predictive of stage transitions at different stages [15].

**Table 1:** Transtheoretical model’s constructs

Constructs	Definitions
Stages of change	
Precontemplation	Not intending to initiate a program of effective stress management in the next six months
Contemplation	Intending to initiate a program of effective stress management in the next six months
Preparation	Intending to initiate a program of effective stress management in the next 30 days
Action	Practicing a program of effective stress management for less than six months
Maintenance	Practicing a program of effective stress management for six months or longer
Experiential processes of change	
Consciousness-raising	Increasing awareness about managing stress
Dramatic relief	Reacting emotionally to warnings about the consequences of not managing stress
Environmental re-evaluation	Considering how the practice or lack of stress management impacts others
Self-re-evaluation	Realizing that managing stress can enhance one’s self-identity
Social liberation	Acknowledging how society is changing to encourage the practice of stress management
Behavioral processes of change	
Self-liberation	Committing to engaging in managing stress
Stimulus control	Restructuring one’s environment to facilitate the process of stress management
Counter-conditioning	Substituting new and positive behavioral choices in the process of managing stress
Helping relationships	Listing and utilizing support resources for managing stress
Reinforcement management	Using positive reinforcement and rewards for undertaking the process of stress management
Pros	The advantages of effective stress management
Cons	The disadvantages of effective stress management
Self-efficacy	The confidence that the individual can engage in effective stress management processes despite any barriers to that process

It is important, however, to note that important critiques of the TTM have been made. In [16], Sutton criticized the definition of each stage in periods because periods such as six months and 30 days were considered arbitrary. In addition, in [17], West argued that the TTM hypotheses about psychological variables, such as pros and cons, and processes causing stage progression were rather vague. Despite these criticisms, in [18], Armitage suggested that such stages of change may represent a useful construct for the segmentation of people into groups, while continuous scales are not preferable and noted that processes of change can offer useful insights to determine intervention targets. If different processes of change, self-efficacy, pros, and cons are found to arguably occasion stage progression at different stages of change for effective stress management, this provides some evidence for the utility of the model’s use of stage classification and the other constructs deployed for understanding its initiation and maintenance. Therefore, it is necessary to examine this point.

A survey of the literature indicated that most previous studies have been cross-sectional [19–28]. Only a few longitudinal studies, however, have been conducted. In [29], Nakamura examined stage transitions over one week in a sample of 99 Japanese college students. In [29], Nakamura observed that some participants progressed from the pre-Action stages to Maintenance, and these transitions could not technically occur because progrssion from the pre-Action stages to Miantenance needs at least six months.These results suggested the difficulty of evaluating the length during which the respondents had engaged in the process of stress management behavior. In [30], Gökbayrak et al. compared processes of change, pros, cons, and self-efficacy across three groups, namely successful changers, relapsers, and non-changers using a sample of 427 American adults who were found not to practice effective stress management. The participants were classified into successful changers, relapsers, and stable non-changers, based on stage transition patterns mapped over 18 months. Comparisons were made at the baseline, and 6, 12, and 18 months later. Successful changers and stable non-changers showed clear differences in terms of five experiential and five behavioral processes, pros, and self-efficacy, most of which tended to be greater in later assessments. Relapsers displayed intermediate scores between the two groups. These results suggested that processes of change, pros, and self-efficacy are associated with stage transitions, somewhat supporting the utility of the stage classification. In their analyses, however, the pre-Action stages and Action and Maintenance were combined. It remains to be explored whether different variables are predictive of stage transitions at different stages in the processes required for effective stress management.

It seems pertinent to examine the utility of the stage classification of the TTM applied to effective stress management throughout eastern Asia including China. This is because the stage classification involves at least two possible challenges as detailed as follows. First, the distinction between Action and Maintenance seems somewhat challenging, as suggested by the results of [29]. Second, a meaningful distinction between daily activities and stress management behavior also seems somewhat challenging. In China, the basic unit of mental health care is the family. Talking with others about stress and seeking help from or giving advice to other family members are profoundly incorporated into daily life so deeply that such stress management behavior per se might not be so easily evaluated.

The purposes of this longitudinal study were twofold. The first was to observe whether there would be cases that showed stage misclassifications over one month. These instances included individuals who progressed from the pre-Action stages to Maintenance over one month. Based on the findings of [29], it was hypothesized that there would be cases that showed stage misclassification (Hypothesis 1). The second was to examine whether different processes of change, self-efficacy, pros, and cons are associated with the stage transitions for processes of effective stress management at different stages. Based on the findings of [30] that the cons did not differ across stage transition groups and general assumptions about the relative importance of experiential processes at the early stages and behavioral processes at later stages [15], with regard to the second purpose, the following three hypotheses were established; Increased and decreased scores of experiential processes would be associated with the forward stage transitions from Precontemplation and Contemplation and the backward transitions from Contemplation (Hypothesis 2); Increased and decreased scores of behavioral processes would be associated with the forward and backward stage transitions from Preparation, Action, and Maintenance (Hypothesis 3); Increased and decreased scores of pros and self-efficacy would be associated with the forward and backward stage transitions from all stages (Hypothesis 4).

## Method

### Participants and procedures

The present study analyzed data from a larger, longitudinal, web-based study on stress management, exercise, leisure activities, and transportation wherein three surveys were conducted in March (Time1 = T1), April (Time2 = T2), and September 2014. The only inclusion criterion was that participants understand and complete all questionnaires. After the participants gave informed consent, they completed the questionnaires with regard to stress management as well exercise, leisure activities, and transportation. A company in China managed and conducted all survey procedures. The procedures and demographic characteristics of the participants have been reported elsewhere [11, 31]. But, briefly, the participants were 130 college students and 1469 workers. The sample size was not determined based on a priori power analysis but governed by the extent of the research budget. The maximum numbers determined by the research budget were 1600 and 1000 in the first and third surveys, respectively. The response rates for the three surveys were unclear. Most participants were female (51.1%, n = 824), married (65.0%, n = 1040), and with a bachelor’s degree (85.6%, n = 1369). Of the participants, 104 reported that they had no stress at all, and were, therefore, excluded from the analyses. The stage distribution was as follows [11]: 10.8% were adjudged to be in the Precontemplation stage (n = 162), 20.4% were adjudged to be in the Contemplation stage (n = 305), 19.1% were adjudged to be in the Preparation stage (n = 285), 28.8% were adjudged to be in the Action stage (n = 430), and 20.9% were adjudged to be in the Maintenance stage (n = 313). Unfortunately, no information was obtained about whether participants had experienced any stressful life events and/or environmental changes while taking part in the study. The authors had not conducted any interventions for managing stress, but it was unclear whether the participants had participated in stress management interventions provided by others. This study analyzes data of demographic variables, perceived stress, stages of change, processes of change, pros, cons, and self-efficacy at T1 and stages of change at T2.

### Measures

#### Demographics

Pertinent to this study, age (years), sex (male or female), marital status (married or not), occupation (13 choices including businessperson and student), and education (graduated elementary school to possessing a doctoral degree) were assessed.

#### TTM variables

The stagesand processes of change, pros, cons, and self-efficacy for effective stress management was measured using the Chinese versions of Pro-Change’s measures that were found to be reliable and valid [26–28, 32]. The staging algorithm [32] first asked participants whether they had been stressed or not, which was used for the data exclusion as described above. Then, they were asked whether they were managing stress effectively and chose one of the five items representing the five stages of change for effective stress management: (1) No. I have no intention to begin in the next six months.” (Precontemplation); (2) “No. But I intend to begin in the next six months.” (Contemplation); (3) “No. But I intend to begin in the next month.” (Preparation); (4) “Yes. I have been practicing but for less than six months.” (Action); (5) “Yes. I have been practicing for at least six months.” (Maintenance).

In addition, four items assessed characteristics of the processes of effective stress management which the participants who were in Action or Maintenance at T1 practiced. The first item was “Do you practice effective stress management alone or with others?” They chose one of two optional answers that included “alone” or “with others.” The second item was “Do you practice this at home or outside the home?” They chose one of two optional answers that included “at home” or “outside the home.” The third item was “Do you practice this through physical activity such as participation in sports?” They chose one of two optional answers of “yes” or “no.” The fourth item was “Is an activity you practice as effective stress management effective in dealing with stress?” They chose one of two optional answers of “yes” or “no.”

The processes of change measure [28] includes 30 items and consists of two higher-order (experiential and behavioral processes) and 10 first-order factors (10 individual processes). Each participant rated each item on a 5-point Likert scale (1 = Never to 5 = Repeatedly) to reflect how frequently they had used each process over the previous month. Cronbach’s alpha coefficients were 0.90 for experiential processes and 0.90 for behavioral processes. The score of each subscale ranges from 15 to 75.

Pros and cons measures [26] include three items each. Each participant was asked to rate how important each statement of pros or cons of effective stress management was for deciding whether or not they would engage in effective stress management on a five-point Likert scale (1 = not important to 5 = extremely important). The total scores of each of the three items in each subscale were calculated as scores for the pros and cons, respectively. These scores range from 3 to 15.

The original self-efficacy measure [27] is a single scale with 10 items. Each item lists specific situations that may make it difficult to maintain the processes of effective stress management. The degree of confidence in those specific situations was rated using a 5-point Likert scale that ranged from 1 = not at all confident to 5 = very confident. Five items were selected to reduce the participants’ burden. The score ranges from 5 to 25.

#### Perceived stress

The Chinese version of the Perceived Stress Scale was used to assess the level of perceived stress of each participant. This is a 14-item, self-reported scale. Each participant was asked to answer each item using a five-point Likert scale score (0 = never to 4 = very often) that best represented how frequently they had experienced each stressful event over the previous month. Total scores ranged from 0 to 56, and higher scores indicate higher levels of perceived stress. This scale has been reported to be reliable and valid [33].

### Statistical analyses

Statistical analyses were conducted using SPSS 28 for Windows. Significance was set at *p* < 0.05. Effect size estimates were interpreted based on [34] guidelines. Values of *η*^2^ and *φ*^2^ of 0.01, 0.06, and 0.14 were interpreted as small, medium, and large, respectively. Values of *d* of 0.20, 0.50, and 0.80 were interpreted as small, medium, and large, respectively. First, using data at T1, the between-stage differences of processes of change, pros, cons, and self-efficacy were examined using multivariate analysis of variance (MANOVA) and follow-up analyses of variance (ANOVAs) (Table 2). Stage membership was set as independent variable. Tukey follow-up tests were conducted. Next, demographics and T1 questionnaire results were compared between the participants who were retained at T2 and those who had dropped out (Table 3). A series of *t* and *χ*^2^ tests were conducted. Third, the patterns of stage transitions over the study period were examined (Table 4). Among 25 possible patterns of stage transitions, 22 patterns could technically occur. In contrast, three patterns would be impossible and these included transitions from Precontemplation, Contemplation, and Preparation to Maintenance. These transitions need six months or longer. Participants who showed these technically impossible transitions were excluded from the data of the participants who completed both the T1 and T2 assessments. Fourth, correlations between the variables were calculated (Table 5). Fifth, six logistic regression analyses were conducted to examine whether experiential processes, behavioral processes, pros, cons, and self-efficacy would be associated with the forward and backward stage transitions (Table 6). Because progression from Action to Maintenance needs a time frame of six months, Action and Maintenance were, therefore, combined. Because it is not possible to examine all 22 stage transition patterns due to the limited number of participants analyzed, stage transitions were, therefore, aggregated into forward, stable, and backward. For example, transitions from Precontemplation to Contemplation, Preparation, Action, and Maintenance were aggregated into the forward transitions. Similarly, those from Preparation to Precontemplation and Contemplation were aggregated into the backward transitions. In the analyses, the independent variables were *T1 scores of* experiential processes, behavioral processes, pros, cons, self-efficacy, and perceived stress. The dependent variables were: (1) whether the stage transition was stable (coded as 0) or forward (coded as 1); (2) whether the stage transition was stable (coded as 0) or backward (coded as 1). These analytic schemes were determined based on the suggestion of [35] that predictors should be measured *before* stage transitions occur for testing the TTM variables as valid predictors of stage transitions.

**Table 2:** Between-stage differences of processes of change, pros, cons, and self-efficacy (*N* = 1495)

Variables	Stage of change for effective stress management					*F*(4, 1490)	*η*_p_^2^	Post hoc comparisons
	PC	C	PR	A	M	(*p* value)		(*p* < .05)
Experiential processes	57.0 ± 12.85	63.0 ± 9.09	63.6 ± 9.60	66.8 ± 7.73	68.2 ± 8.52	48.52 (.00)	.12	PC < C, PR < A, M
Behavioral processes	57.3 ± 12.74	62.7 ± 9.45	62.8 ± 9.90	67.1 ± 7.83	68.0 ± 8.52	48.90 (.00)	.12	PC < C, PR < A, M
Pros	10.9 ± 2.55	11.5 ± 2.13	11.6 ± 2.20	11.6 ± 2.16	12.0 ± 1.96	7.44 (.00)	.02	PC < All; C < M
Cons	8.9 ± 2.42	8.3 ± 2.31	8.7 ± 2.37	8.3 ± 2.67	7.7 ± 2.78	7.26 (.00)	.02	M < All
Self-efficacy	11.8 ± 3.75	12.1 ± 3.51	12.2 ± 3.60	13.9 ± 3.92	14.6 ± 3.97	31.27 (.00)	.08	PC, C, PR < A, M

Values show means ± SDs

PC: Precontemplation; C: Contemplation; PR: Preparation; A: Action; M: Maintenance

**Table 3:** Comparisons of demographics and questionnaire results between participants who were retained and who dropped out

	Retained	Dropped out	*t* or *χ*^2^	*d* or φ^2^
Variables	(*n* = 946)	(*n* = 549)	*(p value)*	
Age in years	32.2 ± 7.47	29.5 ± 7.20	*t* (1493) = 6.92 (.00)	*d* = .37
% Male	48.9	50.1	*χ*^2^ (1) = 0.18 (.67)	φ^2^ = .00
% Married	72.3	53.2	*χ*^2^ (1) = 56.02 (.00)	φ^2^ = .04
% Students	4.7	13.7	*χ*^2^ (1) = 38.49 (.00)	φ^2^ = .03
% Bachelor’s or higher degree	85.3	78.5	*χ*^2^ (1) = 11.29 (.00)	φ^2^ = .01
Perceived stress	31.2 ± 5.58	31.1 ± 5.55	*t* (1493) = 0.10 (.92)	*d* = .01
Experiential processes	65.3 ± 9.56	63.6 ± 10.04	*t* (1493) = 3.15 (.00)	*d* = .17
Behavioral processes	65.3 ± 9.51	63.2 ± 10.48	*t* (1057.15) = 3.77 (.00)	*d* = .21
Pros	11.6 ± 2.13	11.6 ± 2.29	*t* (1493) = 0.34 (.74)	*d* = .02
Cons	8.4 ± 2.56	8.3 ± 2.57	*t* (1493) = 0.76 (.45)	*d* = .04
Self-efficacy	13.1 ± 3.91	13.1 ± 3.95	*t* (1493) = 0.13 (.90)	*d* = .01

Values show means ± SDs

**Table 4:** Stage transition patterns over one month

	Stage at T2						
		PC	C	PR	A	M	Total
**Stage at T1**	PC	27	40	11	8	**8**	94
	C	9	67	43	41	**15**	175
	PR	6	62	53	51	**21**	193
	A	5	49	62	107	48	271
	M	5	32	22	49	105	213
	Total	52	250	191	256	197	946

Numbers in bold indicate stage transitions that cannot technically occur. T1: Time1; T2:Time2; PC: Precontemplation; C: Contemplation; PR: Preparation; A: Action; M: Maintenance

**Table 5:** Correlations between studied variables (N = 902)

Variables	1	2	3	4	5	6
1. Experiential processes		.87^**^	.46^**^	.12^**^	.34^**^	-.44^**^
2. Behavioral processes			.44^**^	.13^**^	.34^**^	-.42^**^
3. Pros				.09^**^	.18^**^	-.26^**^
4. Cons					.10^**^	-.16^**^
5. Self-efficacy						-.20^**^
6. Perceived stress						

Values represent the correlation coefficients between the studied variables

**p < 0 .01

**Table 6:** Significant predictors in logistic regression predicting the forward and backward stage transitions

	Variables	Exp(ß)	95% *CI* Lower–upper	Wald	*p*
Precontemplation	Experiential processes	1.05	1.01–1.09	6.43	.01
Contemplation					
Forward transition	None				
Backward transition	None				
Preparation					
Forward transition	None				
Backward transition	None				
Action/maintenance	Self-efficacy	0.93	0.89–0.98	8.00	< .01

## Results

### Cross-sectional analyses of between-stage differences of the TTM variables

Table 2 shows the scores of studied variables by the stages. The MANOVA for T1 scores resulted in a significant main effect for the stage of change (Wilks’s λ = 0.80, F (24, 3113.03) = 8.81, *p* < 0.01, *η*^2^ = 0.06). Follow-up ANOVAs resulted in significant effects of the stage of experiential processes (*F* (4, 1490) = 48.52, *p* < 0.01, *η*^2^ = 0.12), behavioral processes (*F* (4, 1490) = 48.90, *p* < 0.01, *η*^2^ = 0.12), pros (*F* (4, 1490) = 7.44, *p* < 0.01, *η*^2^ = 0.02), cons (*F* (4, 1490) = 7.26, *p* < 0.01, *η*^2^ = 0.02), and self-efficacy (*F* (4 *F* (4, 1490) = 31.27, *p* < 0.01, *η*^2^ = 0.08). Effect size estimates ranged from small to medium. Results of Tukey follow-up tests are summarized in Table 2.

Most participants in Action and Maintenance practiced effective stress management alone (66.5%, *n* = 494), at home (59.2%, *n* = 440), and through physical activity (89.5%, *n* = 665). With regard to perceived effectiveness, most participants felt that specific activity they engaged in to manage stress was effective (94.9%, *n* = 705).

### Comparisons of demographics and baseline questionnaire results between participants who completed T2 assessment and those who did not

Among 1495 participants, 946 participants completed both T1 and T2 assessments and the remaining 549 participants dropped out. Table 3 indicates the differences in demographics and baseline questionnaire results between participants who completed T2 assessment and those who did not. When compared to the participants who dropped out, the retained participants were older, more likely to be married, less likely to be a student, and more likely to have a bachelor’s or higher degree, and scored significantly higher on experiential processes and behavioral processes.

The percentages of participants who practiced effective stress management alone, at home, and through physical activity were 66.1%, 60.3%, and 89.7% in the retained group and 67.2%, 57.1%, and 89.2% in the dropout group. A series of *χ*^2^ tests i ndicated that both groups had compatible percentages with regard to being alone or with others (*χ*^2^ (1) = 0.09, *p* = 0.77, *φ*^2^ = 0.00), at home or outside home (*χ*^2^ (1) = 0.71, *p* = 0.40, *φ*^2^ = 0.00), and through physical activity or not (*χ*^2^ (1) = 0.04, *p* = 0.84, *φ*^2^ = 0.00). With regard to efficacy, 95.7% of the participants in Action or Maintenance at T1 in the retained group and 93.4% in the dropout group considerd that specific activity they engaged in to manage stress was effective. No significant difference was found (*χ*^2^ (1) = 1.72, *p* = 0.19, *φ*^2^ = 0.00).

### Stage transition patterns

Stage transition patterns over the study period are shown in Table 4. The stage distribution at T1 of 946 participants was as follows: 9.9% in Precontemplation (*n* = 94), 18.5% in Contemplation (*n* = 175), 20.4% in Preparation (*n* = 193), 28.6% in Action (*n* = 271), and 22.5% in Maintenance (*n* = 213). One month later, at T2, the stage distribution was as follows: 5.5% in Precontemplation (*n* = 52), 26.4% in Contemplation (*n* = 250), 20.2% in Preparation (*n* = 191), 27.1% in Action (*n* = 256), and 20.8% in Maintenance (*n* = 197).

Table 4 shows that 25 stage transition patterns were observed. Cases in which participants progressed from the pre-Action stages to Maintenance were checked. Eight participants who were in Precontemplation at the first survey, 15 who were in Contemplation, and 21 who were in Preparation showed these patterns. These patterns could not technically occur. Data of these 44 participants was excluded from the following analyses. Data of the remaining 902 participants was analyzed in later analyses. A series of t and *χ*^2^ tests were conducted to examine differences of demographics and questionnaire scores between the participants who were analyzed (*n* = 902) and those who were excluded (*n* = 44) (data not shown). The participants who were analyzed further were more likely to be male (*χ*^2^ (1) = 3.99, *p* = 0.046, *φ*^2^ = 0.00). Both groups of participants showed compatible scores on perceived stress (*t* (44.93) = 0.39, *p* = 0.70, *d* = 0.09), experiential processes (*t* (944) = 0.99, *p* = 0.32, *d* = 0.15), behavioral processes (*t* (944) = 1.55, *p* = 0.12, *d* = 0.24), and self-efficacy (*t* (944) = 0.82, *p* = 0.41, *d* = 0.13), as well as being fairly matched with regard to marital status (*χ*^2^ (1) = 2.09, *p* = 0.15, *φ*^2^ = 0.00), education (*χ*^2^ (1) = 0.04, *p* = 0.84, *φ*^2^ = 0.00), the student/worker ratio (*χ*^2^ (1) = 2.25, *p* = 0.13, *φ*^2^ = 0.00), and age (*t* (944) = 0.89, *p* = 0.37, *d* = 0.14). The participants who were analyzed further scored significantly higher on pros (*t* (944) = 2.02, *p* = 0.04, *d* = 0.31) and cons (*t* (944) = 2.08, *p* = 0.04, *d* = 0.32).

### Processes, pros, cons, and self-efficacy as predictors of the stage transitions

Correlations between the studied variables are shown in Table 5. Six logistic regression analyses were conducted to identify predictors of the forward stage transitions from Precontemplation, Contemplation, and Preparation and those of the backward stage transitions from Contemplation, Preparation, and Action/Maintenance. Results of these analyses are summarized in Table 6. The results of omnibus tests were found to be significant for the overall regression models predicting the forward transitions from Precontemplation (χ2(1) = 7.15, *p* = 0.01) and the backward transitions from Action/Maintenance (χ2(1) = 8.25, *p* < 0.01). None of the model's variables was predictive of the forward transitions from Contemplation and Preparation and of the backward transitions from Contemplation and Preparation.

The first analysis focused on the forward stage transitions from Precontemplation. Data of 86 participants was analyzed. The forward transitions from Precontemplation was predicted by experiential processes (Exp(*ß*) = 1.05, 95% C.I. 1.01−1.09, Wald(1) = 6.43, *p* = 0.01). Behavioral processes, pros, cons, self-efficacy, and perceived stress were not entered as significant predictors. The participants who used experiential processes more frequently were more likely to progress to the later stages than remain at Precontemplation. The model’s accuracy of the classification was 66.7%.

The second analysis focused on the forward stage transitions from Contemplation. The data of 151 participants were analyzed. No variable was entered as a significant predictor. These variables included experiential processes, behavioral processes, pros, cons, self-efficacy, and perceived stress. The model’s accuracy in terms of classification was 55.6%.

The third analysis focused on the backward stage transitions from Contemplation. Data of 76 participants were analyzed, 67 and 9 of who showed stable and backward stage transitions. No variable was entered as a significant predictor. These variables included experiential processes, behavioral processes, pros, cons, self-efficacy, and perceived stress. The model’s accuracy in terms of classification was 88.2%.

The fourth analysis focused on the forward stage transitions from Preparation. Data of 104 participants was analyzed. No variable was entered as a significant predictor. These variables included experiential processes, behavioral processes, pros, cons, self-efficacy, and perceived stress. The model’s accuracy in terms of classification was 51.0%. 

The fifth analysis focused on the backward stage transitions from Preparation. The data of 121 participants were analyzed. No variable was entered as a significant predictor. These variables included experiential processes, behavioral processes, pros, cons, self-efficacy, and perceived stress. When perceived stress was entered as a predictor, it was not a significant predictor (*p* = 0.06). The model’s accuracy in terms of classification was 56.2%.

The sixth analysis focused on the backward stage transitions from Action and Maintenance. The data of 484 participants were analyzed. The backward stage transitions from Action and Maintenance were predicted by self-efficacy (Exp(ß) = 0.93, 95% C.I. 0.89−0.98, Wald(1) = 8.00, *p* < 0.01). Experiential processes, behavioral processes, pros, cons, and perceived stress were not significant predictors. The participants with lower self-efficacy were more likely to show the backward stage transitions from Action and Maintenance. The model’s accuracy in terms of classification was 64.5%.

## Discussion

The first purpose of this study was to observe whether there would be cases that showed stage misclassification over one month. The participants analyzed were 946 Chinese students and adults. An average participant was young, female, married, and working. Hypothesis 1, which stated that there would be cases that showed stage misclassification, was supported. The results of this study indicated that of 144 participants who progressed from the pre-Action stages to the post-Action stages, 44 showed the progression from the pre-Action stages to Maintenance. The interval was only one month between two assessments, and they could not have progressed to Maintenance. These findings were in line with those of [29], which reported that all five participants who progressed from the pre-Action stages to the post-Action stages showed an unexpected stage transition to Maintenance. The current results provided new information by examining stage transition patterns in a relatively large sample. Up to 30% of participants who progressed to the post-Action stages misclassified themselves into Maintenance. These results suggest the limitations of the model’s stage classification for understanding the process of initiating and maintaining effective stress management.

A relatively high portion of the stage misclassification may be caused by the difficulty of measuring time for stress management. For an accurate judgment of whether one is Action or Maintenance, the individual needs to measure time in the following two ways. First, one needs to evaluate whether they practice effective stress management for at least 20 min or not, requiring one to pay attention to ones's schedule. Second, one also needs to evaluate whether the length of practicing effective stress management is six months or not, requiring one to make a distinction between six months and six months minus one day. It may be necessary to reconsider how the time frame of six months is dealt with. For example, one choice may be the removal of the time frame. Another may be to ask participants to objectively record physical activity. Most participants exercised to manage stress in this sample. This objectively measured physical activity may be useful for clarifying when the individual has initiated effective stress management and how long he has maintained it.

The second was to examine whether different processes of change, self-efficacy, pros, and cons are associated with the stage transitions for effective stress management at different stages. Hypothesis 2, which stated that increased and decreased scores of experiential processes would be associated with the forward stage transitions from Precontemplation and Contemplation and the backward transitions from Contemplation, was rejected. Overall, scores of expreriential processes were not associated with stage transitions. However, one exception was the association of increased scores with the forward stage transitions from Precontemplation. Hypothesis 3, which posited an association of increased and decreased scores of behavioral processes with the forward and backward stage transitions from Preparation, Action, and Maintenance, was rejected. Hypothesis 4, which held that increased and decreased scores of pros and self-efficacy would be associated with the forward and backward stage transitions from all stages, was rejected. Overall, scores of pros and self-efficacy were not associated with stage transitions. However, one exception was that its decreased score was associated with the backward stage transitions from a combined stage of Action and Maintenace.

The results of this study provided new evidence supporting the utility of stage classification of the model applied to effective stress management. Gökbayrak et al. [30] found differences of processes of change, pros, and self-efficacy across three groups, namely, successful changers, relapsers, and non-changers. They combined the pre-Action stages. In addition, they did not examine which of these variables would be associated with the stage transitions. In contrast, this study sub-divided the pre-Action stages into Precontemplation, Contemplation, and Preparation and examined all variables simultaneously as predictors of stage transitions. The sub-division of the pre-Action stages helped us see that experiential processes were only effective in a section of invididuals in Precontemplation. In addition, simultaneous examinations of all variables as predictors helped us see that only experiential processes and self-efficacy were most important in Precontemplation and a combined stage of Action and Maintenance, respectively. These results are in line with the model’s assumption, which states that the different processes of change, pros, cons, and self-efficacy are assumed to be predictors of stage trasitons at different stages. This is important. The stage classification is the most eye-catching aspect of the model [18]. Distinctions across the stages are arbitrary. Critiques of this arbitrariness have been made [16]. If the same set of variables is consistently important in predicting stages transitions at all stages, it reduces the utility of the model’s stage classification [36].

The finding that this study failed to identify variables associated with stage transitions from Contemplation and Preparation suggests the limitation of the model’s variables as predictors of stage transitions. It is important to explore variables associated with stage transitions from Contemplation and Preparation. This is because, if no variable is associated with stage transitions from these stages, it challenges the utility of the model’s stage classification. One possible factor that affects these stages might be how emotionally distressed each participant is. Emotionally distressed individuals may be motivated to buffer emotional distress, which can let them engage in effective stress management. However, this study assessed how stressed they were, but the study did  not assess how emotionally distressed they were. This explanation is a hypothesis, and needs to be examined.

This study also found the inability of behavioral processes and pros to correlate with stage transitions. These results may be explained by the ease of progression to Action, the stress-reducing property of effective stress management, and the interdependent nature of the Chinese culture. In the later part of this paper, these results are discussed with a typical participant, who exercised alone at home to manage stress, as an example. First, it seems relatively easy to initiate effective stress management. Participants exercise at their own schedule and pace at home to make progression through stages. Enhanced use of self-liberation, counter-conditioning, and stimulus control might not be necessary for a transient initiation of effective stress management. Second, effective stress management has the property of reducing distress. This property may make reinforcement management and pros less important. Reinforcement management involves, for example, the presence of another person who praises one when managing stress. Pros refer not to the benefits of effective stress management but the relative importance of merits for determining whether one engages in effective stress management or not. Over 90% of the participants in Action or Maintenance felt that the stress management behavior they engaged in certainly reduced stress. Individuals easily experience distress-reducing properties by a transient initiation of effective stress management. This experience itself can motivate and reinforce or increase the probability of practicing effective stress management. Third, happiness in Eastern cultures such as China is associated with good relationships with others to a greater extent than in Western cultures such as the United States [37, 38]. It is common for Chinese people to support each other. For example, an essential unit of mental health care in China is family. A source of support is more likely to be friends and family than professionals. In line with this, a substantial percentage of participants (33.5%) practiced effective stress management with others in this study. During daily life, people commonly praise and support each other. This mutually related culture may make helping relationships and reinforcement management less important.

### Practical implications

The present results have practical implications for intervention developments if replicated robustly. Increased use of experiential processes was found to predict the forward stage transitions only from Precontemplation. Its use can be facilitated by psychological interventions. Such interventions include, for example, providing information about stress management and societal change where stress management is more encouraged and making individuals privy to the knowledge that stress management can improve self-image. It is suggested that such interventions might be more effective for those in Precontemplation than they would in Contemplation and Preparation.

Decrease of self-efficacy was found to predict the backward stage transitions from a combined stage of Action and Maintenance. Self-efficacy can also be enhanced by psychological interventions. It was proposed by Bandura [39] as being enhanced, for example, by successful past experiences, observing successful experiences of others, and modeling their behaviors. Applied to effective stress management, successful experiences might be facilitated by helping an individual to choose a relatively easy but still effective way to manage stress. Observing others who practice effective stress management and modeling their behaviors might be facilitated by providing the opportunity to learn what others do to manage stress.

### Limitations

While the results of this study extended the previous findings of the utility of stage classification associated with stage transitions, this study was limited in the following points. First, due to the limited number of participants, this study did not examine all 22 stage transition patterns. Stage transitions were broadly aggregated into forward, stable, and backward. This aggregation may have overlooked the predictive ability of processes of change, pros, cons, and self-efficacy of stage transitions. This is because these variables might be importantat when progressing from one stage to the next stage. Second, scores of both experiential and behavioral processes were high. This means that the participants of this study worked hard for practicing effective stress management. It remains unclear whether these scores are limited to the present sample or not. Thus, the generazability of the findings is unclear. Despite of these limitations, results of this study provided a rationale to conduct a next study which further examines the utility of stage classification associated with stage transitions.

## Conclusions

Results provided only limited support for the utility of the stage classification and clarified the limitations of it as a tool. Higher experiential processes and lower self-efficacy predicted the forward and backward stage transitions from Precontemplation and Action/Maintenance. No variables predicted the stage transitions from Contemplation and Preparation. Of 144 participants who progressed from the pre-Action stages to the post-Action stages, 44 then progressed to Maintenance. These patterns could not technically occur. These misclassification cases provided evidence to suggest the limitations of the stage classification.

## Data Availability

The dataset of this study is available from the corresponding author on reasonable request.

## Abbreviation

TTM: Transtheoretical model
